# Professional Plagiarism

**Published:** 1891-04

**Authors:** 


### Editorial.

### PROFESSIONAL PLAGIARISM.

It is questionable whether the majority of readers of dental literature have any conception of the amount of plagiarism of ideas existing in the profession, and it may be surmised that very little interest has been felt, from the entire absence of any allusion to it.

Dentistry has been an outgrowth of innumerable experiments. The foundation was laid in the mechanism of the past. The workers of metals, from the earliest history of the race, had their share in its formation. The artificers in the precious metals that made Italy famous, that gave birth to a Benvenuto Cellini during the Renaissance, with his wonderful art-productions in gold and silver; the Chinese, with their porcelain, and its rediscovery in Europe by Böttger, in Meissen, Germany, all, and more, had to do with the building of our own profession.

Starting from this, dentistry was laid upon new and untried lines. Experience was added to experience, experiment to experiment, failure to failure, and success to success, until a firm basis was secured. Who can tell to-day who were the originators of these many forms of work? Where are the names recorded that should be known and honored? Who can answer?

For a profession barely one hundred and fifty years old, this neglect is certainly not a creditable showing. The excuse may be given that each member has been too busy making professional history to stop to record it. This is only in part true.

The real cause lies not so much in records as it does in the fact that ideas have been appropriated in part and by wholesale in books, papers read before societies, and in practice, until the question of the origin is lost in dire confusion. The unscrupulous character of this work is apparent to the close observer.

One of the most insidious means of destroying all original reputation is the plan adopted by some writers of books. They will ostentatiously parade names of authors in their preface from whom selections have been made, and then deliberately extract ideas by wholesale and ingeniously weave these in with their own in a way that defies recognition, thus depriving the originals of all credit. Another class will take a portion of an investigator's work and attach it to personal ideas and send this out as original.

Another individual will scan the various journals for ideas and make note of them. In time he is called upon to prepare a paper for some society, and these are then harmoniously blended by the skilful scribe into an essay to be received with honor by his fellows.

Still another discards all these ingenious modes to make a reputation without the trouble of earning it, and boldly opens the journal or encyclopædia and *copies* such of the productions as may suit, *verbatim et literatum.*

This picture is. not overdrawn, nor would it be difficult to illustrate by known cases.

Renewed attention has been called recently to this subject by the fact that original work done by the editor of this journal twenty-six years ago, and subsequently published in several articles, and finally in the "System of Dentistry," had been copied from the latter and republished in a monthly dental journal as an original production. It was then transferred to another publication, and the credit in both instances given to the plagiarist. In this case no attempt was made to change the words or construction. Having copied it literally, it was read before a prominent dental society, and was, it is said, received with favor.

From a perhaps mistaken regard for the youth of the individual, this case is alluded to only in general terms. It is cited here, therefore, not as a personal grievance, but as a professional wrong and typical of that moral obliquity which prevails in regard to such matters, and which is certainly sapping the life of our calling.

The impossibility of having a large number of men brought together for special work without a proportion of dishonest ones among them seems to render it necessary that a place of record should be provided, where new ideas and plans of procedure may be entered. If the American Dental Association would undertake this, and publish the results yearly, it would be invaluable in the present, and settle many disputed questions in the future.

No one who entertains a real interest in the growth of his profession cares for a moment what may become of the few grains of original work he may be able to scatter in his lifetime, but he naturally feels a repugnance to have his neighbor transfer the product to his own garden and call the plant by his own name.

The remarkable success which attended the effort of the young man in the case alluded to, in that it could pass the members of a society and two astute editors, is a warning that should not go unheeded. It calls for closer reading and the memorizing of original data. This, of course, means a labor which very few will care to undertake, and the difficulty can only be met by the plan proposed,—a place of record.

